# The hemispheric lateralization of speech processing depends on what “speech” is: a hierarchical perspective

**DOI:** 10.3389/fnhum.2012.00309

**Published:** 2012-11-16

**Authors:** Jonathan E. Peelle

**Affiliations:** Department of Otolaryngology, Washington University in St. LouisSt. Louis, MO, USA

A recurring question in neuroimaging studies of spoken language is whether speech is processed largely bilaterally, or whether the left hemisphere plays a more dominant role (cf., Hickok and Poeppel, 2007; Rauschecker and Scott, 2009). Although questions regarding underlying mechanisms are certainly of interest, the discussion unfortunately gets sidetracked due to the imprecise use of the word “speech”: by being more explicit about the type of cognitive and linguistic processing to which we are referring it may be possible to reconcile many of the disagreements present in the literature.

## Levels of processing during connected speech comprehension

**Figure 1:**
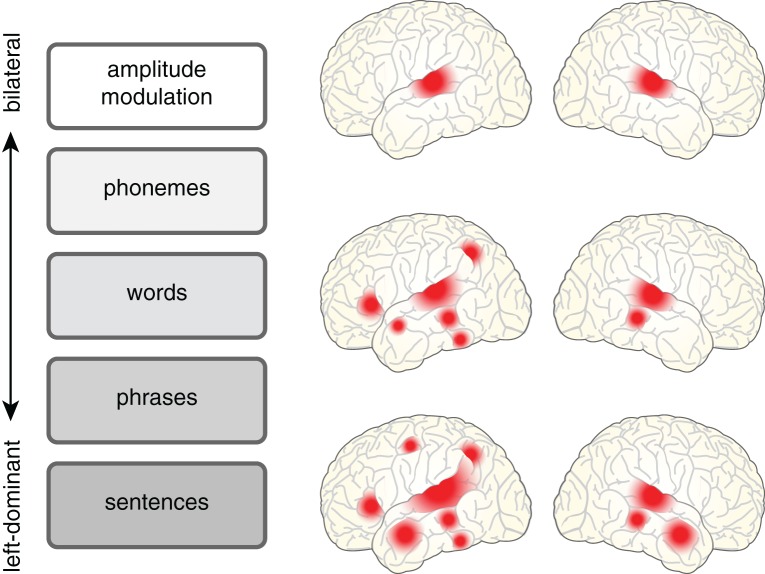
**The cortical regions involved in processing spoken language depend in a graded fashion on the level of acoustic and linguistic processing required.** Processing related to amplitude modulated noise is bilateral (e.g., Giraud et al., 2000), shown at top. However, as the requirements for linguistic analysis and integration increase, neural processing shows a concomitant increase in its reliance on left hemisphere regions for words [see meta-analysis in Davis and Gaskell (2009)] and sentences [see meta-analysis in Adank (2012)].

A relatively uncontroversial starting point is to acknowledge that understanding a spoken sentence requires a listener to analyze a complex acoustic signal along a number of levels, listed schematically in Figure 1. Phonemes must be distinguished, words identified, and grammatical structure taken into account so that meaning can be extracted. These processes operate in an interactive parallel fashion, and as such are difficult to fully disentangle. Such interdependence also means that as researchers we often use “speech” as a term of convenience to mean:

1. Amplitude-modulated noise or spectral transitions, as might be similar to aspects of spoken language;
2. Phonemes (“b”), syllables (“ba”), or pseudowords (“bab”);
3. Words (“bag”);
4. Phrases (“the bag”);
5. Sentences (“The bag of carrots fell to the floor”) or narratives.

Naturally, because different types of spoken language require different cognitive mechanisms—spanning sublexical, lexical, and supralexical units—using an unqualified term such as “speech” can lead to confusion about the processes being discussed. Although this point might seem obvious, a quick review of the speech literature demonstrates that many authors^1^ have at one time or another assumed their definition of “speech” was obvious enough that they need not give it, leaving readers to form their own opinions.

Below I will briefly review literature in relation to the neural bases for two types of spoken language processing: unconnected speech (isolated phonemes and single words) and connected speech (sentences or narratives). The goal is to illustrate that, within the context of a hierarchical neuroanatomical framework, there are aspects of “speech” processing that are both bilateral and lateralized.

## Unconnected speech is processed largely bilaterally in temporal cortex

The first cortical way station for acoustic input to the brain is primary auditory cortex: not surprisingly, acoustic stimuli activate this region robustly in both hemispheres, whether they consist of pure tones (Belin et al., 1999; Binder et al., 2000) or amplitude-modulated noise (Giraud et al., 2000; Hart et al., 2003; Overath et al., 2012). Although there is speculation regarding hemispheric differences in specialization for these low level signals (Poeppel, 2003; Giraud et al., 2007; Obleser et al., 2008; McGettigan and Scott, 2012), for the current discussion, it is sufficient to note that both left and right auditory cortices respond robustly to most auditory stimuli, and that proposed differences in hemispheric preference relate to a modulation of this overall effect^2^.

Beyond low-level acoustic stimulation, phonemic processing requires both an appropriate amount of spectral detail and the relationship to a pre-existing acoustic category (i.e., the phoneme). The processing of isolated syllables results in activity along the superior temporal sulcus and middle temporal gyrus, typically on the left but not the right (Liebenthal et al., 2005; Heinrich et al., 2008; Agnew et al., 2011; DeWitt and Rauschecker, 2012). Although this may suggest a left hemisphere specialization for phonemes, listening to words (which, of course, include phonemes) reliably shows strong activity in bilateral middle and superior temporal gyrus (Price et al., 1992; Binder et al., 2000, 2008). In addition, stroke patients with damage to left temporal cortex are generally able to perform reasonably well on word-to-picture matching tasks (Gainotti et al., 1982); the same is true of healthy controls undergoing a Wada procedure (Hickok et al., 2008). Together these findings suggest that the right hemisphere is able to support at least some degree of phonemic and lexical processing.

That being said, there are also regions that show increased activity for words in the left hemisphere but not the right, particularly when pseudowords are used as a baseline (Davis and Gaskell, 2009). Both pseudowords and real words rely on stored representations of speech sounds (they share phonemes), but real words also involve consolidated lexical and/or conceptual information (Gagnepain et al., 2012). Left-hemisphere activations likely reflect the contribution of lexical and semantic memory processes that are accessed in an obligatory manner during spoken word recognition. Within the framework outlined in Figure 1, spoken words thus lie between very low level auditory processing (which is essentially bilateral) and the processing of sentences and narratives (which, as I will discuss below, is more strongly left lateralized).

Processing of phonemes and single words therefore appears to be mediated in large part by both left and right temporal cortex, although some indications of lateralization may be apparent.

## Connected speech relies on a left-lateralized frontotemporal network

In addition to recognizing single words, comprehending connected speech—such as meaningful sentences—depends on integrative processes that help determine the syntactic and semantic relationship between words. These processes rely not only on phonemic and lexical information, but also on prosodic and rhythmic cues conveyed over the course of several seconds. In other words, a sentence is not simply a string of phoneme-containing items, but conveys a larger meaning through its organization (Vandenberghe et al., 2002; Humphries et al., 2006; Lerner et al., 2011; Peelle and Davis, 2012). In addition to providing content in and of itself, the syntactic, semantic, and rhythmic structure present in connected speech also supports listeners' predictions of upcoming acoustic information.

An early and influential PET study of connected speech by Scott et al. showed increased activity in the lateral aspect of left anterior temporal cortex for spoken sentences relative to unintelligible spectrally-rotated versions of these sentences (Scott et al., 2000). Subsequent studies, due in part to the use of a greater number of participants, have typically found intelligibility effects bilaterally, often along much of the length of superior temporal cortex (Crinion et al., 2003; Friederici et al., 2010; Wild et al., 2012a). In addition, a large and growing number of neuroimaging experiments show left inferior frontal involvement for intelligible sentences, either compared to an unintelligible control condition (Rodd et al., 2005, 2010; Awad et al., 2007; Obleser et al., 2007; Okada et al., 2010; Peelle et al., 2010a; McGettigan et al., 2012; Wild et al., 2012b) or parametrically correlating with intelligibility level (Davis and Johnsrude, 2003; Obleser and Kotz, 2010; Davis et al., 2011). Regions of left inferior frontal cortex are also involved in processing syntactically complex speech (Peelle et al., 2010b; Tyler et al., 2010; Obleser et al., 2011) and in resolving semantic ambiguity (Rodd et al., 2005, 2010, 2012; Snijders et al., 2010). In most of these studies activity in right inferior frontal cortex is not significant, or is noticeably smaller in extent than activity in the left hemisphere. These functional imaging studies are consistent with patient work demonstrating that participants with damage to left inferior frontal cortex have difficulty with sentence processing (e.g., Grossman et al., 2005; Peelle et al., 2007; Papoutsi et al., 2011; Tyler et al., 2011).

Processing connected speech thus relies more heavily on left hemisphere language regions, most obviously in inferior frontal cortex. The evidence outlined above suggests this is largely due to the increased linguistic demands associated with sentence processing compared to single words.

## The importance of statistical comparisons for inferences regarding laterality

In many of the above papers (and in my interpretation of them) laterality was not statistically assessed, but inferred based on the presence or absence of an activation cluster in a particular brain region. That is, seeing a cluster of activation in left inferior frontal gyrus but not the right, and concluding that this particular task has a “left lateralized” pattern of neural activity. However, simply observing a response in one region, but not another, does not mean that these regions significantly differ in their activity (the “imager's fallacy”; Henson, 2005). This is a well-known statistical principle, but one that can remain difficult to follow in the face of compelling graphical depictions of data (Nieuwenhuis et al., 2011).

Nevertheless, for true claims of differential hemispheric contributions to speech processing, the left and right hemisphere responses need to be directly compared. Unfortunately, for functional imaging studies hemispheric comparisons are not as straightforward as they seem, in part because our left and right hemispheres are not mirror images of each other. There are, however, a number of reasonable ways to approach this challenge, including:

1. Extracting data from regions of interest (ROIs), including independently defined functional regions (Kriegeskorte et al., 2009) or probabilistic cytoarchitecture (Eickhoff et al., 2005), and averaging over voxels to compare left and right hemisphere responses. Sometimes these ROIs end up being large, which does not always support the specific hypotheses being tested, and not all regions may be available. However, this approach is relatively straightforward to implement and interpret.
2. Using a custom symmetric brain template for spatial normalization (Bozic et al., 2010). This may result in less veridical spatial registration, but enables voxel-by-voxel statistical tests of laterality by flipping images around the Y axis, avoiding the problem of ROI selection (and averaging).
3. Comparing left vs. right hemisphere responses using a multivariate classification approach (McGettigan et al., 2012). Multivariate approaches are robust to large ROIs, as their performance is typically driven by a smaller (more informative) subset of all voxels studied. Multivariate approaches may be somewhat more challenging to implement, however, and (depending on the size of the ROI used) may limit spatial specificity.

In the absence of these or similar statistical comparisons, any statements about lateralization of processing need to be made (and taken) lightly.

## Conclusions

I have not intended to make any novel claims about the neural organization of speech processing, merely to clarify what has already been shown: phonological and lexical information is processed largely bilaterally in temporal cortex, whereas connected speech relies on a left-hemisphere pathway that includes left inferior frontal gyrus. Importantly, the distinction between unconnected and connected speech is not dichotomous, but follows a gradient of laterality depending on the cognitive processes required: lateralization emerges largely as a result of increased linguistic processing.

So, is speech processed primarily bilaterally, or along a left-dominant pathway? It depends on what sort of “speech” we are talking about, and being more specific in our characterizations will do much to advance the discussion. Of more interest will be future studies that continue to identify the constellation of cognitive processes supported by these neuroanatomical networks.

## Footnotes

^1^Including me.

^2^In fact, the term “lateralization” is also used to variously mean (a) one hemisphere performing a task and the other not being involved, or (b) both hemispheres being engaged in a task, but one hemisphere is doing more of the work or being slightly more efficient, potentially compounding the confusion.
